# Apoptosis: Reprogramming and the Fate of Mature Cells

**DOI:** 10.5402/2012/685852

**Published:** 2012-04-17

**Authors:** Hoi-Hung Cheung, Xiaozhuo Liu, Owen M. Rennert

**Affiliations:** Section on Clinical and Developmental Genomics, The Eunice Kennedy Shriver National Institute of Child Health and Human Development, National Institutes of Health, Bethesda, MD 20892, USA

## Abstract

Apoptosis is essential for embryogenesis, organ metamorphosis, and tissue homeostasis. In embryonic stem cells, self-renewal is balanced with proliferative potential, inhibition of differentiation, and prevention of senescence and apoptosis. Growing evidence supports the role of apoptosis in self-renewal, differentiation of pluripotent stem cells, and dedifferentiation (reprogramming) of somatic cells. In this paper we discuss the multiple roles of apoptosis in embryonic stem cells (ESCs) and reprogramming of differentiated cells to pluripotency. The role of caspases and p53 as key effectors in controlling the generation of iPSC is emphasized. Remarkably, the complication of apoptosis arising during reprogramming may provide insights into technical improvements for derivation of iPSC from senescent cells as a tool for modeling aging-related diseases.

## 1. Introduction

Apoptosis is a type of programmed cell death first described by Kerr et al. in the 1970s. It is morphologically characterized by cell shrinkage, membrane blebbing and nuclear condensation, and formation of apoptotic bodies [1]. The apoptotic process is initiated by “death” signals, which trigger a complex series of events with multiple positive and negative feedback loops [2]. A central step in the execution of the apoptotic process is the activation of caspases, a group of enzymes belonging to the cysteine protease family. Activation of caspases cleaves many vital cellular proteins, breaking down the nuclear scaffold and cytoskeleton, and subsequently leads to nuclear DNA degradation [3].

Based on the original source of “death” signals, caspase-dependent apoptosis is classified into two pathways: intrinsic pathway, which is activated by modulators within the cell itself, and extrinsic pathway, which responds mainly to extra-cellular stimuli [4]. In the extrinsic pathway (also known as receptor-mediated death pathway), death ligands such as TRAIL and FasL induce apoptosis by activating the death receptors (DR4/TRAILR1/Apo2 and Fas/Apo1, etc.) at the cell surface. The activated death receptors oligomerize, which leads to recruitment of the adaptor proteins such as FADD (Fas-associated protein with death domain) and further chain activation of caspase-8 and −3 [2, 4, 5]. The intrinsic pathway, which is also known as mitochondria-mediated death pathway, is initiated by damage to mitochondria which results in the release of a series of proteins into cytoplasm, including cytochrome c. When released into the cytoplasm, cytochrome c complexes with Apaf-1, and this complex further activates caspase-9 and ultimately caspase-3. Activated caspase-3 further cleaves critical intracellular proteins and thereby induces the final stage of cell death [4–6].

A critical event for the intrinsic pathway is release of cytochrome c from mitochondria, a step strictly regulated by members of the B-cell lymphoma 2 (Bcl-2) family [6, 7]. Members of this family are divided into two subfamilies: one includes the proapoptotic proteins such as Bak, Bax, and Bid; another includes the antiapoptotic proteins such as Bcl-2 and Bcl-xL [5]. Recent studies suggest that induction of pro-apoptotic proteins and inhibition of anti-apoptotic proteins, triggered by certain anti-cancer agents, directly alter the permeability of mitochondrial membrane and further induce the apoptosis signaling pathway by release of cytochrome c and/or SMAC/Diablo [5, 8].

Apart from the Bcl-2 family, p53 protein, a well-known tumor suppressor protein, is also a key player in apoptosis. p53 can “sense” DNA damage and trigger a transient cell cycle arrest to allow DNA repair. In the situation of extensive DNA damage, p53 will promote irreversible cell cycle arrest (senescence) or apoptosis [9]. Therefore, p53 is considered as a key guardian of the genome [10]. p53 can induce both the intrinsic and extrinsic apoptotic pathways [11]. It has been reported to be involved in the caspase-8-dependent intrinsic pathway [12]. In addition to direct activation of Bax, p53 can trigger caspase-8-dependent Bid cleavage, which results in Bax conformation and induces cytochrome c release into the cytosol from mitochondria [13].

Collectively, apoptosis is essential for the sophisticated architecture of life including normal cell turnover, organ metamorphosis, tissue homeostasis, and embryonic development [7, 14–17]. Here we briefly introduce the general background of apoptosis. The details of the molecular players participating in apoptosis have previously been reviewed [3, 18, 19].

In this paper we briefly summarize some recent studies on apoptotic events found in embryonic stem cells (ESCs), and link them to the cellular reprogramming, with the emphasis on the role of p53 and caspases, that reverses differentiated cells to an undifferentiated state. We also discuss the crosstalk between reprogramming, accelerated aging, and senescence.

## 2. Apoptosis in Human Embryonic Stem Cells

Human embryonic stem cells (hESCs) have evoked more and more interest from biomedical and developmental researchers since the first hESC line was derived in 1998, as these pluripotent cells provide an opportunity to develop essential treatments for currently incurable diseases; in addition cultured hESCs offer a useful in vitro system to study the processes of human development [20–22].

hESCs, the pluripotent cell population derived from the blastocyst, are characterized by unlimited self-renewal and pluripotency, the ability to differentiate into all cell types representing all three germ layers both in vitro and in vivo. Stem cells are at higher risk of accumulating potentially deleterious mutations than differentiated somatic cells since stem cells have a higher potential for self-renewal [7, 23]. Inadequate genomic stability is particularly deleterious for hESCs capacity for self-renewal and organismic development, since they are the progenitors of all organ systems, including germ cells [23]. Therefore, hESCs have a more stringent mechanism to ensure their genomic stability and integrity than differentiated cells. There is some evidence to support this postulation, including the following: (1) lower mutation frequency (such as errors in replication, point mutation) is observed in ESCs than in somatic cells; (2) abnormal ESCs with DNA damage are removed from the self-renewal pluripotent population through apoptotic or differentiation programs [23, 24]. As a guardian of the genome, p53 has been shown to be a critical component, but its role in the induction of apoptosis is independent of its classic action in nuclear accumulation and induction of cell cycle arrest [25–29]. For example, in response to DNA damage, p53, in most somatic cells, may induce cell cycle arrest at G1 through activation of its downstream target genes (e.g., p21), whereas, in ESCs, p53 cannot trigger the G1 checkpoint [24]. Qin et al. demonstrated that, in DNA-damaged hESCs, p53 cannot transactivate its downstream target Mdm2. The lack of Mdm2 induction disrupts the feedback degradation circuitry and promotes an overaccumulation of p53. The high level of p53 induces spontaneous apoptosis through the mitochondrial pathway to remove these abnormal stem cells, as well as spontaneous differentiation by suppressing pluripotency genes Oct-4 and Nanog. When hESCs differentiate, the ability of p53 to activate the transcription of its target genes is restored [26]. In summary, apoptosis serves as a protective role in hESCs by eliminating stem cells that are abnormal and potentially dangerous from the pluripotent population [30].

hESCs, unlike mouse ESCs, are more vulnerable to apoptosis following cellular detachment and single-cell dissociation [31]. Such fragility upon dissociation has been a challenge for maintaining and developing hESC for biological research or for medical applications. After complete single-cell dissociation, hESCs undergo massive cell death, and the cloning efficiency of dissociated cells is generally less than 1% [32]. The apoptosis following cellular dissociation may be caused by disruption of the hESC microenvironment (i.e., niche), which is a consequence of alteration of signaling factors, cell-cell interactions (e.g., E-cadherin pathway), and cell-ECM interactions (e.g., integrin pathway). The niche is implicated in playing a pivotal role in hESC survival and self-renewal. Sasai’s group demonstrated that, following dissociation, membrane expression of E-cadherin is irreparably disrupted, leading to activation of the ROCK (Rho-associated kinase) pathway, particularly hyperactivation of actomyosin [32–34]. In addition, they showed that Y-27632 (a ROCK inhibitor) and Blebbistain (a myosin inhibitor) efficiently suppress dissociation-caused apoptosis. Moreover, Ding’s group identified additional small molecules, Thiazovivin and Pyrintegrin markedly promote hESC survival after trypsin dissociation and enhance adhesion to matrigel- or laminin-coated plates (ECM-coated plates), but not gelatin-coated plates. These compounds enhance cell-ECM adhesion and activate the integrin signaling pathway, which acts synergistically with growth factors to promote cell survival [34]. Collectively, these findings imply that ESCs maintain self-renewal through different functions of cell adhesion pathways and may explain the fundamental differences in morphology, growth factor requirement, and sensitivity to enzymatic cell dissociation.

In addition, maintenance of self-renewal capacity is critical for hESC preservation. The transcription factors, such as Oct4, Sox2, Nanog, and c-Myc, have essential roles in maintaining self-renewal. They form a regulatory circuitry of autoregulation and feedforward loops [35–37]. Interference with this regulatory circuitry may lead to apoptosis, differentiation, or cell senescence in ESC cultures. A recent study of hESCs demonstrated that sustained activation of the oncogene c-Myc induces apoptosis via activation of caspase and triggers differentiation by reduction of Oct4 and Nanog expression [38].

In this section, we discussed the different apoptotic features of hESCs, with emphasis on the roles in genome integrity maintenance and the role in single-cell-dissociation-induced apoptosis (i.e., ROCK-dependent hESCs apoptosis). We hope these observations will improve culturing techniques for hESCs and lead to maintenance of self-renewal with minimal genetic instability, an essential requirement for the development of hESCs for human therapeutics.

## 3. Apoptosis: Somatic Cell Reprogramming and the Role of p53

**Figure 1:**
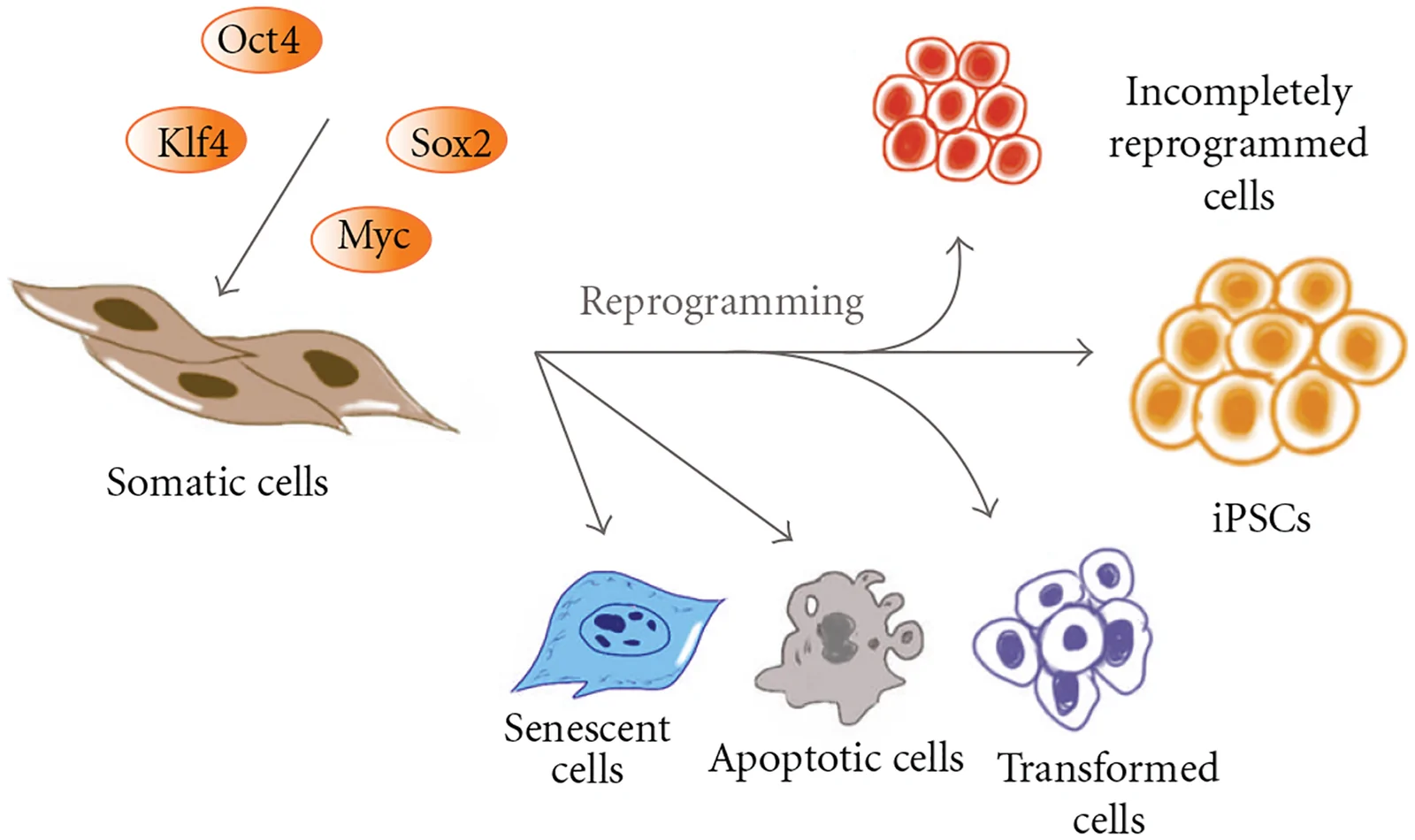
Different cell fates arisen from introduction of reprogramming factors. Overexpression of Oct4, Sox2, Klf4, and c-Myc results in generation of apoptotic cells, senescent cells, transformed cells, incompletely reprogrammed cells, and ESC-like iPSCs. During the course of induction of pluripotency, many cells undergo apoptosis or become senescent. A large proportion of cells remained in culture represent incompletely reprogrammed or become transformed.

Reprogramming of differentiated cells into the pluripotent, undifferentiated state is achieved by expression of three or four transcription factors, Oct4, Sox2, Klf4 (OSK), and c-Myc (OSKM) [39]. The in vitro derived cells, called iPSCs, are dedifferentiated and resemble ESCs in many ways, including expression profiles and epigenetic status, albeit differences exist [40, 41]. Reprogramming towards pluripotency is a slow, stochastic process characterized by a large proportion of incompletely reprogrammed cells, and extensive cell death during the process of induction (Figure 1) [42]. Apoptosis occurs in the first few days following expression of the introduced reprogramming factors, and this phenomenon may contribute to the low efficiency of iPSC generation.

The critical role of apoptosis during somatic cell reprogramming is highlighted by the participation of p53 and the induction of apoptotic caspases by the 3- or 4-factor combination. As a crucial protein for tumor suppression, p53 plays different roles in senescence, apoptosis, cell cycle arrest, and DNA repair. Expression of c-Myc, one of the Yamanaka factors, shows the most dominant effect on induction of p53 and the downstream p21. However, omission of c-Myc and induction by OSK 3-factor may also activate p53, suggesting a common transcriptional network leading to cellular stress activates the p53-dependent pathway.

Early experiments generating iPSC with 4-factors was inefficient (~0.05%), especially for human skin fibroblasts. Zhao et al. first demonstrated the role of p53 on the suppression of reprogramming efficiency [43]. RNAi knockdown of p53 improved the efficiency of iPSC generation by ~100-fold, even in the absence of c-Myc. Subsequently several important papers verified that p53 was a critical factor limiting iPSC induction [44–47]. Data from Belmonte’s group demonstrated that knockdown of p53 or its target gene, p21 (also known as Cdkn1a), significantly increases reprogramming efficiency [45]. Based on the observation that the 3-factor cocktail induces the expression of the pro-apoptotic factor Bax (a p53-inducible gene), antagonizing reprogramming-induced apoptosis will also increase the number of iPSC colonies. Overexpression of Bcl2 (a Bax antagonist) suppresses apoptosis regardless of whether induction is initiated by two, three, or four reprogramming factors. Moreover, repression of p19^Arf^ and p16^Ink4a^ increases iPSC formation. p19^Arf^ stabilizes p53 by inhibiting Mdm2, whereas p16^Ink4a^ inhibits phosphorylation of the retinoblastoma gene product (Rb) by cyclin D-dependent kinases; reduction of both these factors rescues cell cycle arrest and improves reprogramming efficiency. Serrano’s group found that the Ink4/Arf locus is epigenetically silenced in iPSC, but upregulated in aged cells, suggesting an important role of the Ink4a/Arf locus as an epigenetic barrier to reprogramming [48].

Similar experiments from Blasco’s group support the role of p53 in activation of the DNA damage response and apoptosis in a p53-dependent mechanism. p53 is critically involved in the regulation of cell cycle arrest and apoptosis by preventing the propagation of DNA-damaged cells, including those with short or dysfunctional telomeres. Using genetically modified MEF cells that lack telomerase (Terc^−/−^), they showed a significant proportion (10% and 15%) of wild-type cells undergo apoptosis, whereas it is further increased to 40% in G3 Terc^−/−^ cells at days 9–13 after infection. Knockdown of p53 or overexpression of Bcl2 abrogates apoptosis at the onset of iPSC induction, allowing cells with pre-existing damaged or dysfunctional telomeres to reprogram [46]. These studies indicate that p53 limits reprogramming by inducing apoptosis at the time of pluripotency induction. Consistent with other investigators, Yamanaka’s group showed the involvement of the p53-p21 pathway as a barrier to iPSC generation. In the p53-null background, induction efficiency is boosted to 10%. Even terminally differentiated T lymphocytes can be successfully reprogrammed [44].

Li et al. documented the important finding of caspase involvement during reprogramming [49]. Two key apoptotic proteases, caspases 3 and 8, are activated following transduction of the reprogramming factors (c-Myc was excluded in their experiments because it is able to activate caspases and induce apoptosis). Oct4, the indispensable factor for iPSC generation, is responsible for activation of caspases 3 and 8. In agreement with Blasco’s group, they found 10–20% of cells undergo apoptosis following OSKM factor induction. However, in the absence of c-Myc using the OSNL factors, apoptosis is reduced to 1–3%, suggesting a critical role of c-Myc in the activation of apoptosis. The activation of caspases during reprogramming eliminates cells by apoptosis, meanwhile benefits iPSC induction, as shown by a significant reduction of iPSC formation after knockdown of caspases 3 or 8. The importance of caspase activation in promoting reprogramming is demonstrated by its capacity to degrade Rb, which is a tumor suppressor gene that regulates cell cycle progression and helps the promotion and maintenance of cellular differentiation. It appears that the Rb and p53 pathways work independently to suppress iPSC formation; however, the exact mechanism remains to be uncovered.

Reprogramming involves extensive chromatin remodeling and induction of the pluripotency network to reset the epigenome to an ESC-like state [50]. During the process reactive oxygen species (ROS) are generated by metabolism [51]. Increased levels of ROS leads to DNA damage, cell senescence, and apoptosis. ROS may hinder survival of reprogramming cells, as suggested by observations of enhancement of iPSC generation during hypoxia [47, 52]. In addition, mitochondrial content and oxidative stress are repressed in iPSCs or hESCs [53], suggesting that ROS generation by reprogramming factors is unfavorable for generating iPSCs. Esteban et al. identified vitamin C as an effective chemical to boost iPSC generation; however surprisingly the underlying mechanism seems not totally dependent on its antioxidant activity [51]. Instead, treatment of vitamin C reduces p53/p21 levels, which is the main barrier to successful reprogramming. They further identified histone demethylases Jhdm1a/1b as the downstream effectors of vitamin C [54]. In particular, Jhdm1b promotes cell cycle progression and suppresses senescence by repressing the Ink/Arf locus during reprogramming. Furthermore, inhibition of the mTOR pathway by rapamycin or PP242 or insulin/IGF-1 signaling pathway notably enhances the efficiency of reprogramming [55]. From the concept that reprogramming is a stressful process that activates apoptosis and cellular senescence, targeting the mTOR pathway alleviates senescence imposed by DNA damage response [56]. These findings support the role of senescence as a limiting factor for successful reprogramming.

**Figure 2:**
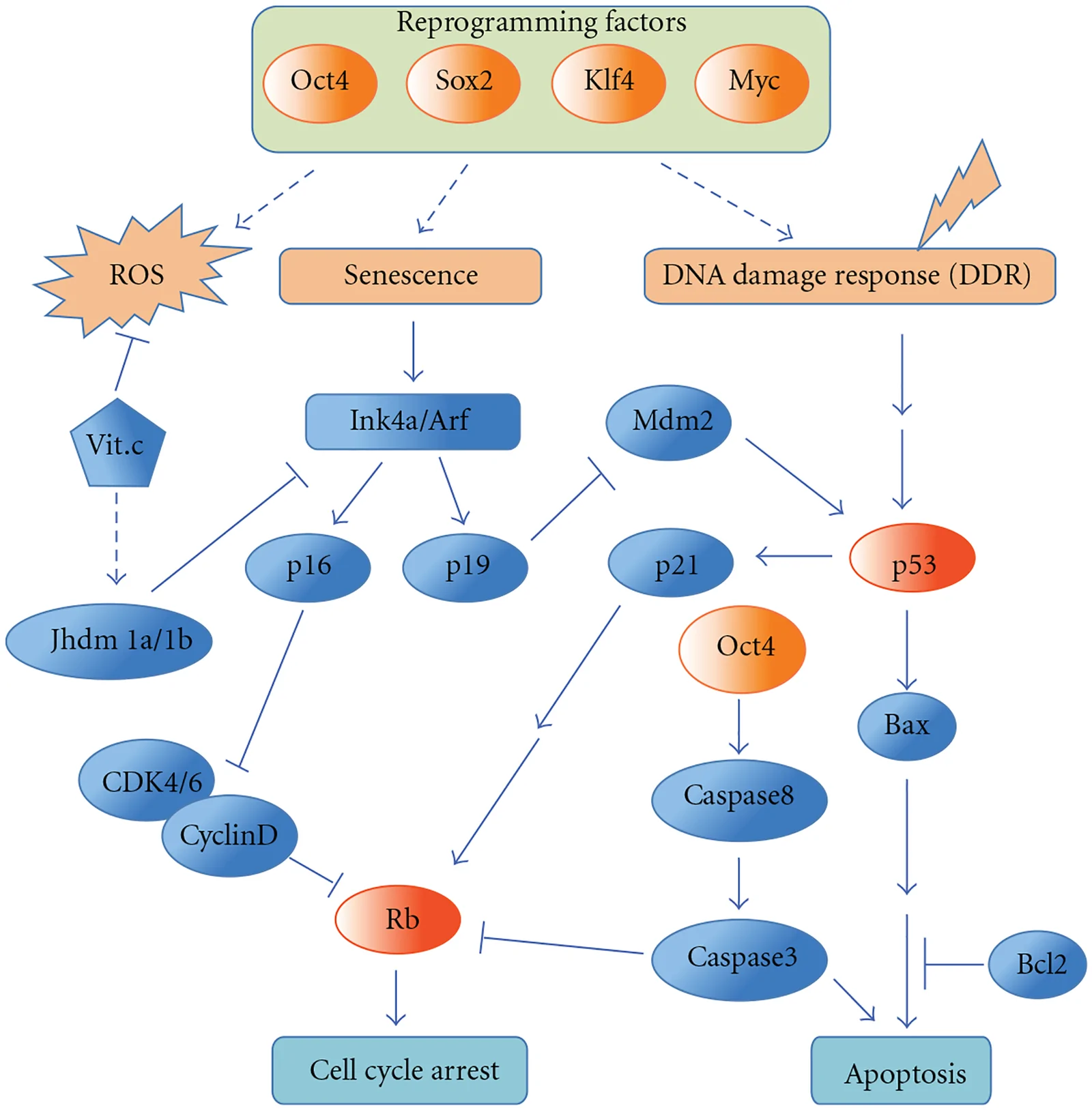
The critical role of p53 tumor suppressors in limiting the generation of iPSC by induction of apoptosis or cell cycle arrest. Cell reprogramming is a stressful process accompanied with generation of ROS, induction of senescence, and DNA damage response. Activation of p53 and its downstream p21 observed during reprogramming is likely induced by DNA damage response. p21 is a cyclin-dependent kinase (CDK) inhibitor that regulates cell cycle arrest. Besides, senescence-induced expressions of p16 and p19 indirectly activate tumor suppressors p53 and Rb, respectively, leading to cell cycle arrest and apoptosis. Oct4-dependent activation of caspase 8 and 3 has a dual role in promoting apoptosis and cleavage of Rb, which favors reprogramming. Treatment of cells with Vitamin C also facilitates iPSC formation by scavenging ROS and meanwhile regulates histone demethylases Jhdm1a/1b, the latter of which is able to promote cell cycle progression and suppress senescence by repressing the Ink4/Arf locus. Double arrows: multiple steps.

In summary, introduction of reprogramming factors appears to exert a cellular stress that will lead to DNA damage, senescence, and apoptosis. Activation of p53 and other apoptotic players is a protective mechanism that eliminates damaged or senescent cells from continuing replication, a similar mechanism that p53 does by suppressing tumorigenesis (Figure 2).

## 4. Cellular Senescence, Accelerated Aging, and Reprogramming

Transcription-factor-based reprogramming results in generation of different fates of somatic cells. The reprogramming factors can trigger cellular senescence and apoptosis, leading to cell death [53]. For those cells that can bypass the stress, the majority form transformed cells, partially reprogrammed iPSCs, and very low percentage of cells form true iPSCs. The Ink4a/Arf locus is epigenetically silenced in iPSC or ESC, but expressed in differentiated fibroblasts [46]. Ink4a/Arf expression is associated with natural aging and with passage number in culture [46, 47]. Activation of the Ink4a/Arf locus is found during reprogramming and becomes a rate-limitation in iPSC generation. This observation is consistent with the upregulation of the p16^Ink4a^ and p19^Arf^ in aged fibroblasts, which display a lower reprogramming efficiency. p19^Arf^ and p16^Ink4a^ regulate p53 and Rb tumor suppressors respectively, both of them are important players in cell cycle progression and proliferation.

Reprogramming of aged or senescent cells becomes technically challenging [57], given the fact that these cells show slow rates of proliferation, shortening of the telomere, increased DNA damage, and upregulation of p53 and Ink4a/Arf genes. MEF cells from the third-generation mice deficient in telomerase carry accumulated short telomeres and can barely be reprogrammed [58]. However, this defect can be ameliorated by reintroduction of telomerase or suppression of p53 [46, 58]. Remarkably, in p53^−/−^ background, iPSC derived from telomerase-deficient MEF cells show even further shortening of telomeres, suggesting that inhibition of p53 technically bypasses the p53-dependent cell death, but does not “rejuvenate” the reprogrammed iPSCs with elongated telomeres [46]. In humans, the shortened telomere is generally correlated with aging. Despite that, human iPSCs have been successfully derived from skin biopsies of old patients of 82 and 89 years old, although the efficiency of iPSC generation was not comparable to young donors [59]. Recently, senescent fibroblasts derived by prolonged culture (51 PDs) or fibroblasts directly taken from centenarians were reprogrammed with a six-factor cocktail (OSKMNL). The derived iPSCs reset telomere length and are indistinguishable from normal hESCs [60]. Interestingly, additional reprogramming factors NANOG and LIN28 are essential for generating iPSC for these senescent cells, and a longer time of induction is required, implicating certain intrinsic senescence barriers that need to be overcome by these additional factors.

Reprogramming differentiated cells to iPSC is analogies to rejuvenation of “aged” cells to their ground state, allowing these pluripotent cells to redifferentiate into different lineages. An alternative excellent model for the study of senescence and reprogramming potentially is the premature aging syndromes derived from patients who display accelerated aging phenotypes such as premature grey hair (or alopecia), arteriosclerosis, heart disease, and shortened lifespan. The Hutchinson-Gilford progeria syndrome (HGPS) is a segmental progeroid syndrome that occurs in children with dominant mutation of the LMNA gene. The aberrantly spliced form of the LMNA gene product (progerin) causes irregular nuclear organization and loss of functional integrity of the nuclear lamina. Accumulation of DNA damage (double-strand breaks and abnormal DNA damage response) and cell cycle arrest are commonly found in HGPS cells [61]. Secondly, progerin induces premature aging by increasing DNA damage signaling and activation of p53 and Rb pathways. Progerin-induced DNA damage signaling is localized to telomeres and induces dysfunction of telomeres, indicative of an accelerated aging phenotype in HGPS cells [62]. Reprogramming of HGPS skin fibroblasts to iPSC has been reported by different groups [63–65]. The derived HGPS-iPSCs demonstrate absence of progerin expression, normal nuclear structure, and epigenetic markers. However upon differentiation, abnormal cellular phenotypes associated with this syndrome reappear. Zhang et al. further demonstrated that DNA damage and nuclear abnormalities recur in redifferentiated mesenchymal stem cells (MSCs), vascular smooth muscle cells, and fibroblasts, recapitulating the aging phenotype of HGPS [63]. Questions still remain whether these reprogrammed patient iPSCs can properly reset the dysfunctional telomeres to the normal state. Another progeroid syndrome, the Werner syndrome (WS), is an accelerated aging phenotype of adults caused by recessive mutation of the DNA helicase WRN [66]. No iPSCs have been derived from WS patients to date. The lessons we learned about the mechanisms of cellular senescence and reprogramming involving central safe guardians such as p53/Rb and the barrier of senescence like Ink4/Arf and telomere shortening may provide insights into technical refinement for derivation of iPSC from senescent cells and as a tool for modeling aging-related diseases.

## 5. Concluding Remarks and Future Perspectives

In this paper we discussed the multiple roles of apoptosis in ESC and iPSC induction. We highlighted the importance of apoptotic caspases and p53 in the activation of apoptosis and cellular senescence. The knowledge we learn from these recent studies uncovers a previously unknown function of p53 in safeguarding reprogramming in addition to its well-known function in tumor suppression. Here we summarize two important points.

First, understanding the role of apoptosis in ESC and somatic cell reprogramming to iPSC may help us evaluate the current technology aiming at making clinically “safe” iPSC for cell therapy in the future. A major concern of iPSC-based therapy is the tumorigenicity of iPSC, particularly for the risk of overexpressing Klf4 and c-Myc, both of them are known oncogenes [67]. The latter has been found to induce apoptosis and differentiation in human ESC and trigger cellular senescence during reprogramming [38]. Additionally, the pivotal role of p53 activation during reprogramming appears to be a self-defense mechanism that prevents cells from oncogenic transformation. Whereas p53 mutation is commonly found in many cancers, the inhibition of p53 for boosting iPSC generation is highly risky for generating clinical grade iPSC, as demonstrated by Marión et al. that pre-existing DNA damage and dysfunctional telomeres are still present in p53-null iPSC [46]. The similarity between oncogenesis and reprogramming raises concerns about the utilization of these p53-null iPSCs for therapy. Non-transcription-factor-based reprogramming methods by using microRNAs and small molecules as examples are potentially good alternatives, although the safety of these iPSCs needs to be assessed [68]. The future direction is to develop safe protocols for generating clinical grade iPSCs with high efficiency and low risk of tumorigenesis without the need of any genetic modification.

Second, understanding the role of senescence in reprogramming provides a molecular basis for deriving iPSC from aging-related diseases such as HGPS and WS. For instance, to reprogram premature aged somatic cells from WS, inhibition of p53/p21 or Ink4a/Arf may help to overcome the pre-existing senescent barrier and dysfunctional telomeres found in these cells. It is also interesting to understand whether somatic cell reprogramming can properly reset the accumulative epigenetic changes arising from natural aging or premature aging to a normal state. We foresee that reversing senescence in aged cells will have clinical implications for developing cell-based therapy for the treatment of aged individuals and patients of progeria.
